# The effect of bystander cardiopulmonary resuscitation on the survival of out-of-hospital cardiac arrests: a systematic review and meta-analysis

**DOI:** 10.1186/s13049-018-0552-8

**Published:** 2018-10-11

**Authors:** Jianting Song, Wenxiu Guo, Xiaoguang Lu, Xin Kang, Yi Song, Dianbo Gong

**Affiliations:** 1 Dalian Medical University, No. 9, west section, Lushun South road, Dalian city, Liaoning Province China; 2 Liaoning university of Traditional Chinese Medicine, No. 79, Chongshan road, Huanggu District, Shenyang city, Liaoning province China; 3 Department of Emergency Medicine, Affiliated Zhongshan Hospital of Dalian University, No. 6th Jiefang Street, ZhongShan District, Dalian city, Liaoning Province China

**Keywords:** Bystander CPR, Initial rhythm, Out-of-hospital cardiac arrest, Meta-analysis

## Abstract

**Background:**

For many years, bystander cardiopulmonary resuscitation (BCPR) has been considered as a favorable factor to improve survival of out-of-hospital cardiac arrests (OHCAs). To examine the effect of BCPR on the survival of OHCAs and whether BCPR might also improve survival when the initial rhythm of OHCAs is limited, we performed a meta-analysis on published observational studies.

**Methods:**

We did a systematic review to identify all studies published up to March, 2018, in any language, that reported the relation between BCPR and the survival of OHCAs. Using standard forms, two authors independently identified studies for inclusion and extracted information. The outcome was survival. Meta-regression was done to ascertain weighted factors for the outcomes.

**Results:**

Data were extracted from 19 studies involving 232,703 patients. Firstly, pooled odds ratio (OR) from 16 cohort studies showed that BCPR was associated with improved chance of survival of OHCAs compared with NO-BCPR (OR 1.95, 95% confidence interval [CI]: 1.66–2.30). Secondly, from 8 cohort studies of OHCAs whose initial rhythm is limited, the pooled OR was 2.10 (95% CI, 1.68–2.63) of 6 articles for shockable rhythm and 1.07 (95% CI, 0.37–3.13) of 2 articles for non-shockable rhythm. Meta-regression showed a relation between the survival of OHCAs and BCPR was influenced by area (*p* < 0.05).

**Conclusions:**

Based on currently available evidence, the findings of this meta-analysis suggest that BCPR increases the survival of OHCAs, and it also help OHCAs whose initial rhythm is shockable. That is to say BCPR is also helpful when emergency department response time is short. Therefore global priority should be given to increasing the incidence of BCPR by evidence-based best practice.

## Background

Cardiac arrest (CA) is a sudden loss of blood flow resulting from the heart suddenly stops beating, and it usually causes death if not treated within minutes. There are many causes of cardiac arrest, and cardiogenic disease is the most common reason which accounting for 75–85% [1]. Although the survival of cardiovascular diseases has improved significantly over the past 30 years, the survival of out-of-hospital cardiac arrests (OHCAs) is not apparently increasing globally [2]. According to statistics 5 million people worldwide will suffer from out-of-hospital cardiac arrest (OHCA) every year, of which only 7% will survive [3, 4].

Low survival of OHCAs can be attributed to many factors of the chain of survival. Despite years of guidelines of each country for cardiopulmonary resuscitation and emergency cardiovascular care update, bystander cardiopulmonary resuscitation (BCPR) remains the most important factor to improve the survival of OHCAs [5]. Some researchers found that the delay to defibrillation was increased with the development of times, possibly because heavier traffic and the lack of emergency resources [6]. This result made BCPR become a more important link of the the survival chain of OHCA.

In 1973, American Heart Association’s decision to endorse cardiopulmonary resuscitation (CPR) training of the lay public give support to the concept of large-scale training in many regions [7], and then there was a lot of trainings of BCPR successively all over the world. The bystanders of BCPR refers to onlookers of the OHCAs without medical background, which include trained and untrained lay rescuers in our meta-analysis.

This systematic review aimed to summarize current research results on the survival of BCPR for OHCAs, and make further discussion of the mechanism of BCPR to improve the survival by limiting the initial rhythm of OHCAs. and assessed whether BCPR improved the outcome depending upon the initial rhythm of OHCAs.

## Materials and methods

### Search strategy and selection criteria

We followed the proposed MOOSE (Meta-Analysis of Observational Studies in Epidemiology) [8] guidelines to report the present meta-analysis. We conducted a search of published English-language articles through searched PubMed and Embase databases from the data of inception until March, 2018 with search terms like “out-of-hospital cardiac arrest”, “out-of-hospital ventricular fibrillation/ventricular tachycardia/asystole/pulseless electrical activity”, “bystander cardiopulmonary resuscitation”. Additionally, reference lists of every article were screened for further related publications. There were no restrictions on language, publication date or publication status.

Studies would be included if they agreed with the following criteria: 1) cohort design study; 2) containing BCPR and NO-BCPR, were done for adult patients with out-of-hospital cardiac arrest; 3) providing adjusted OR, and corresponding 95% CI, or the number of events that can calculate them.

In addition, studies which designed as case reports, systematic reviews or studies with mutual overlapping populations were excluded from this meta-analysis.

### Data extraction

Two reviewers independently extracted the following data from each eligible study: first author’s last name, year of publication, site of origin, study period, study design, patients’ age, the number of cases and controls, adjusted OR, statistical adjustments of confounding factors. Survival to discharge was the primary outcome variable, if data of survival to hospital discharge were not available, we used 30-day survival as the primary outcome. Any disagreements were resolved by consensus.

### Methodological quality assessment

The risk of bias was analyzed according to Newcastle-Ottawa Scale (NOS) for cohort studies, which consists of three parameters of quality: selection, comparability, and outcome assessment. NOS assigns a maximum score of 4 for selection, 2 for comparability, and 3 for outcome. Hence, a score of 9 is the highest and reflects the highest quality.

### Data synthesis and analysis

Firstly, we computed a pooled OR and 95% CI by using Stata 12.0 to generate forest plots, to determine whether there was a statistical association between BCPR and survival and to assess heterogeneity of studies for all OHCAs. Heterogeneity was quantified evaluated using the chi-square based Cochran’s Q statistic [9] and the I^2^ statistic, this statistic yields results ranging from 0 to 100% (I^2^ = 0–25%, no heterogeneity; I^2^ = 25–50%, moderate heterogeneity; I^2^ = 50–75%, large heterogeneity; and I^2^ = 75–100%, extreme heterogeneity) [10]. If heterogeneity existed, the random effects model was used, otherwise, the fixed effects model was used. In addition, we analyzed which factors influence heterogeneity by meta-regression analysis. If significant heterogeneity is identified, subgroup analysis was also conducted according to the result of meta-regression. If possible, potential publication bias was assessed by visual inspection of the funnel plots of the primary outcome [11]. The Begg rank correlation test was used to examine the asymmetry of the funnel plot [12] and the Egger weighted linear regression test was used to examine the association between mean effect estimate and its variance [11], *P* < 0.05 indicated bias, and *P* > 0.05 indicated no publication bias. All statistical calculations were performed by Stata 12.0.

Secondly, we computed a pooled OR and 95% CI to present the effect of BCPR for patients whose initial rhythms are shockable and non-shockable respectively, and calculated heterogeneity with the method mentioned above. If heterogeneity existed, the random effects model was also used here.

## Results

### Literature search and selection

**Fig. 1:**
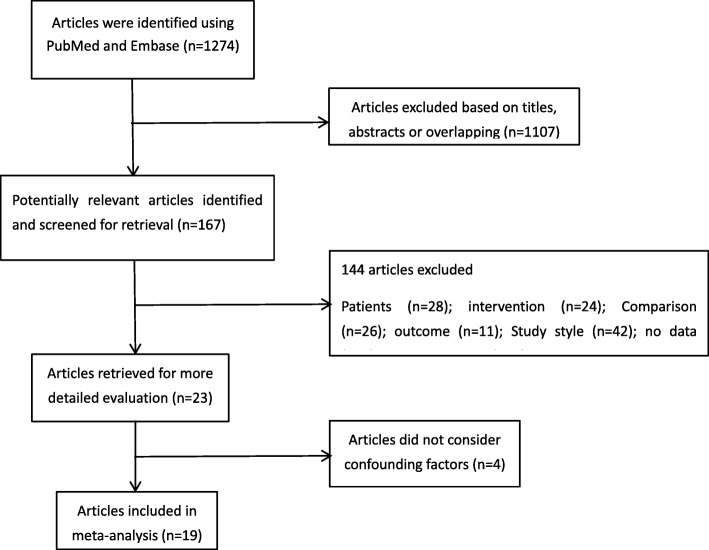
Summary of the studies selection process

The search strategy generated 19 references: PubMed (*N* = 934), Embase (*N* = 340). After 170 duplicates discarded, we excluded publications based on titles and/or abstracts, mainly because they are reviews, animal studies, case reports, editorials, letters, comments and conference abstracts. 167 full-text studies were identified for further assessment. Subsequently, studies were excluded in line with the principle of PICOS (Patients-Intervention-Comparison-Outcome-Study Style). Among these studies, we excluded 144 studies which are mainly irrelevant to the PICOS: the characteristics of patients (28 studies) including only pediatric patients and OHCA with a non-cardiac cause, intervention (24 studies) including the comparison of the use of rescue drugs/the rescue method/the time when the onlookers started CPR/where the cardiac arrest occurred/whether there is a dispatcher-assisted BCPR, comparison (26 studies) including no control group/no relevant comparison, outcome (11 studies) including neurologic outcome/mortality/return of spontaneous circulation, study style (42 studies), studies where data could not be extracted (7 studies), and duplicate reports (6 studies). Since all of our included studies are observational studies, and the survival of OHCAs is the result influenced by multiple factors, considering the confounding factors is very important to study the effect of BCPR. Therefore, this meta-analysis excludes 4 articles that do not consider confounding factors [13–16]. Finally, 19 articles were included. A flow chart showing the study selection is presented in Fig. 1.

### Description of the studies

The characteristics of 19 prospective/retrospective cohort studies are presented in Table 1. These studies were published from 1985 to 2017. Firstly, there are 16 articles studied BCPR for all OHCAs, which include eight studies were conducted in Europe [17–24], 3 in Asia [25–27], 4 in America [28–31], 1 in Oceania [32]. The sizes of the cohorts ranged from 722 to 66,780. There are two ORs in one article [21], one from 1992 to 1995 and the other from 2002 to 2005. Secondly, 8 articles reported OHCA whose initial rhythm were shockable or non-shockable, 6 [6, 24, 25, 29, 32, 33] for shockable rhythm, only 2 [33, 34] for non-shockable rhythm.

**Table 1:** Characteristics of included studies

NO.	Author(year)	Location/Period	Population	Age (yrs)	Mean EMS response time (minutes)	Outcomes	Adjustment for covariates
1	Cummins(1985) [28]	Washington, America (1976.04–1983.09)	OHCAs (1297)	Mean 64.7	N/A	1.survival to hospital discharge	Witnessed arrest, age, sex, and EMS response time
2	Swor(1995) [29]	Oakland, American (1989.06–1993.06)	OHCAs (722) VT/VF (476)	65.9	ALS/BLS 6.5/3.6	1.VT/VF 2. live discharge 3. ROSC 4. survival to hospital admission 5. survival at 1 year	Age, ALS response interval
3	Weston(1997) [17]	Glamorgan, England (1989–1992)	OHCAs (954)	N/A	N/A	1. admission to hospital 2. discharge from hospital	Age, sex, arrival status, cause of arrest, rhythm of arrest and the delay to ALS
4	Stiell(1999) [30]	Ontario (1991.01–1995.01)	OHCAs (5335)	Mean 68	N/A	1. survival to discharge	Age, bystanders witnessed arrest, fire CPR, EMS response time
5	Holmberg(2000) [18]	Sweden (990.01–1995.05)	OHCAs (9877)	Med 70	N/A	1. 30-day survival	Age, location of arrest, witnessed status, time between call to first ECG
6	Finn(2001) [32]	Perth,Australia (1996–1999)	OHCAs (1293) VT/VF (286)	Mean65	N/A	1. initial cardiac arrest rhythm of VF/VT in bystander-witnessed cardiac arrests of presumed cardiac origin 2. 28-day survival	Age, gender
7	Herlitz,(2003) [19]	Goteborg, Sweden (1980.10–2000.10)	OHCAs (5505)	N/A	N/A	1. survival to hospital discharge	age, previous history, chronic medication before cardiac arrest, events at resuscitation, and status on admission to hospital
8	Herlitz(2005) [20]	Sweden (1990–2000)	OHCAs (22024)	Mean71 med 73	Med 6	1. survival to 1 month	age, sex, location of arrest, witnessed status, initial cardiac rhythm, EMS response time
9	Iwami(2007) [25]	Osaka, Japan (1998.05–2003.04)	OHCAs (4902) VT/VF (696)	Mean 62	Mean 9.7	1. neurologically favorable 1-year survival 2. return of spontaneous circulation; admission to hospital; and 1-week, 1-month, and 1-year survival 3. VT/VF	Age, witnessed status, place, defibrillation, EMS response time
10	Herlitz(2008) [34]	Sweden (1990–2005)	OHCAs (22465)	Mean 67	N/A	1. 30-day survival	Age, witnessed status, place, defibrillation, EMS response time
11	Nordberg(2009) [21]	Sweden (1992.01–2005.12)	OHCAs (34125)	Mean 63	Med 7.5	1. 30-day survival 2. VT/VF	Age, sex, witnessed status, initial rhythm, EMS response time
12	Adielsson(2011) [6]	Sweden (1990.01–2009.12)	OHCAs: VT/VF (7187)	N/A	N/A	1. 30-day survival 2. patients who survived to hospital admission	Age, sex, the site of cardiac arrest, Increasing delay to defibrillation
13	Lindner(2011) [22]	Stavanger, Norway (2001.01–2008.12)	OHCAs (846)	Mean 71	Med 8.5	1. ROSC 2. survival to discharge	age, year, witnessed status, initial cardiac rhythm, EMS response time
14	Wissenberg(2013) [23]	Denmark (2001–2010)	OHCAs (19468)	Med 72	Med 11	1. 30-day survival	age, sex
15	Nehme(2015) [33]	Victoria, Australian (2002.07–2012.06)	OHCAs VT/VF (5561)	Med 70	Med 8.0	1. survival to hospital discharge 2. survival to hospital discharge (shockable rhythm)	age, sex, year of arrest, witnessed status, initial cardiac rhythm, EMS response time
16	Lai (2015) [26]	Singapore (2001–2004,2010–2012)	OHCAs (5453)	Mean 62	Med 6.2	1. survival to discharge	age, year, witnessed status, initial cardiac rhythm, EMS response time, Mechanical CPR by EMS, prehospital advanced airway, medicine
17	Hasselqvist-Ax(2015) [24]	Sweden (1990.01–2011.12)	OHCAs (30381) VT/VF (10094)	Med 72	Med 7	1. 30-day survival 2. VT/VF	age, sex, location and cause of cardiac arrest, initial cardiac rhythm, EMS response time, time from collapse to call for EMS, and year of event
18	Gaieski(2017) [31]	Philadelphia (2008–2012)	OHCAs (4625)	Med 64	Med 6.6	1. prehospital ROSC 2. 30-day survival	Age, sex, shockable rhythm, time of arrest, witnessed status, location of cardiac arrest, prehospital AED
19	Tanaka(2017) [27]	Asia (2009.01–2012.12)	OHCAs (56765)	Mean 72.7 Med 76.0	N/A	1. survival to hospital discharge or 30 day hospital survival 2. favorable postarrest cerebral performance	age, bystander-witnessed arrest, shockable rhythm, bystander CPR, out-of-hospital defibrillation, advanced airway and drug administration, and response time

*OHCAs* out-of-hospital cardiac arrests, *VT* ventricular tachycardia, *VF* ventricular fibrillation, *ALS* advanced life support, *BLS* basic life support, *ROSC* restoration of spontaneous circulation, *EMS* Emergency medical services, *Med* median, *N/A* not available

### Quality of the included studies

There was good agreement between the reviewers in regards to the validity assessments, the quality assessment of all the published studies were shown in Table 2. All of the studies were of high quality (NOS score higher than 6). In terms of population selection bias, none of the articles from Africa fitted our inclusion criteria. Selection bias is likely because of unpublished data, abstracts, and presentations were not included. In terms of comparability bias, most studies included adequate matching or adjustments for covariates such as emergency medical services (EMS) response interval and the patients’ age and so on. The most common outcome bias was the lack of blinding.

**Table 2:** Quality assessment according to the Newcastle-Ottawa Scale

Study	Selection	Comparability	Outcome	Total
Cummins(1985)	4	2	2	8
Swor(1995)	4	2	2	8
Weston(1997)	3	2	2	7
Stiell(1999)	3	2	2	7
Holmberg(2000)	3	2	2	7
Finn(2001)	4	1	2	7
Herlitz(2003)	3	1	2	6
Herlitz(2005)	4	2	2	8
Iwami(2007)	4	2	2	8
Herlitz(2008)	3	2	2	7
Nordberg(2009)	3	2	2	7
Lindner(2011)	3	2	2	7
Wissenberg(2013)	4	1	2	7
Lai(2015)	3	2	2	7
Nehme(2015)	3	2	2	7
Hasselqvist-Ax(2015)	4	2	2	8
Gaieski(2017)	4	2	2	8
Tanaka(2017)	4	2	2	8

### Effects of interventions

#### Survival of all OHCAs

**Fig. 2:**
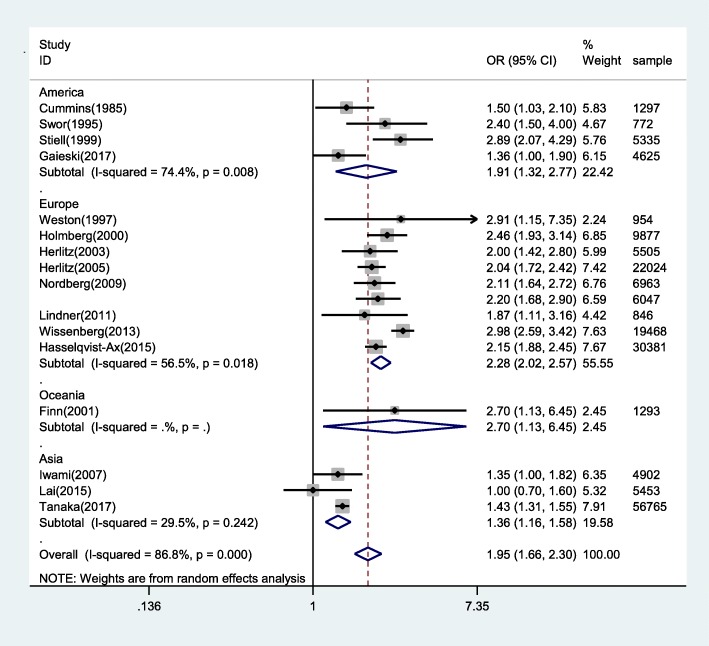
Forest plot of studies reporting BCPR stratified by area

Sixteen studies prospectively or retrospectively investigated the association between BCPR and survival of OHCAs. Meta-analysis of these studies showed a significantly increased chance of survival with BCPR comparing with NO-BCPR (Fig. 2) (OR: 1.95; 95%CI: 1.66–2.30; *P* < 0.05). Substantial heterogeneity was observed (*P* < 0.001; I^2^ = 86.8%)

#### Survival of OHCAs whose initial rhythm is shockable

**Fig. 3:**
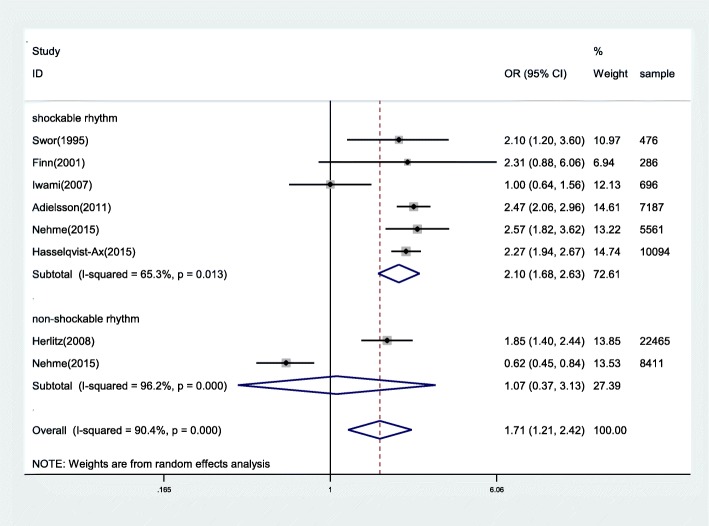
Forest plot of studies reporting BCPR stratified by initial rhythm

6 studies reported the association between BCPR and survival of OHCAs whose initial rhythm was shockable, giving a total sample of 24,300 participants. The data was analyzed by using a random-effects model according to the heterogeneity test result (*P* = 0.013, I^2^ = 65.3%). There was a significant difference between BCPR and NO-BCPR in patients whose initial rhythm is shockable with the OR of 2.10 (95%CI, 1.68–2.63, *P* < 0.05) (Fig. 3).

#### Survival of OHCAs whose initial rhythm is non-shockable

2 studies demonstrated the relationship between BCPR and the survival of OHCAs whose initial rhythm was non-shockable. A random-effects model was also used here. However, the ORs for the association varied from 0.62 to 1.85 across studies. Overall, no difference was recorded between BCPR and NO-BCPR for OHCAs of which initial rhythm is non-shockable (OR: 1.07 [95%CI, 0.37 to 3.13]; *P* > 0.05). Substantial heterogeneity was observed (*P* < 0.001, I^2^ = 96.2%) (Fig. 3).

#### Regression and subgroup analyses

Meta-regression analyses were conducted to assess predictors of heterogeneity among odds ratios. Through multiple meta-regression analyses, area may lead to heterogeneity among the possible heterogeneity factors(years, area, outcome index) (Table 3). Then subgroup analysis was conducted according to regional differences (Fig. 2).

**Table 3:** Meta-regression analysis of Included Studies

VARIABLES	y
Outcome	0.298
	−0.19
Year	−0.00502
	−0.0104
Area	−0.150†
	−0.0636
Constant	10.5
	−20.66
Observations	17

Notes_Titles: ‡ *p* < 0.01, † p < 0.05, * *p* < 0.1

#### Publication Bias

**Fig. 4:**
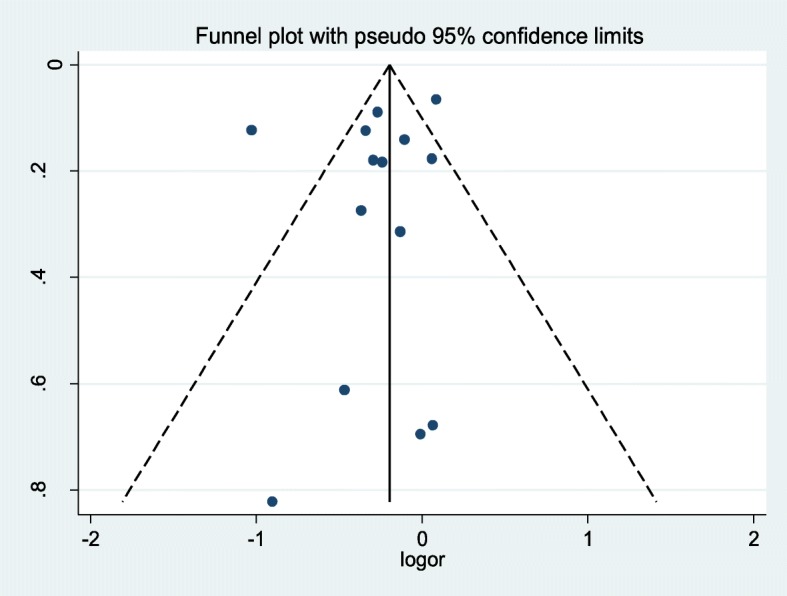
Funnel plot based on Odds Ratio for association between BCPR and the survival of OHCAs

Figure 4 showed that the funnel plot was symmetrical, that indicated there was no publication bias existed. The Begg rank correlation test and Egger linear regression test also indicated no evidence of publication bias among studies of BCPR and the survival of OHCAs. (Begg, *p* = 0.827; Egger, *p* = 0.439)

## Discussions

A timely and effective cardiopulmonary resuscitation can improved survival of OHCAs, and we all know that closed chest CPR can save the patients who suffered from cardiac arrest since 1960s. However, there was no systematic review or meta-analysis evaluating the effect of BCPR by limiting the initial rhythm of OHCAs. The initial rhythm of cardiac arrest is classified into “shockable” versus “non-shockable”, as determined by the electrocardiograph (ECG) rhythm. This refers to whether a cardiac dysrhythmia can be treatable by using defibrillation. The two “shockable” rhythms are ventricular fibrillation(VF) and pulseless ventricular tachycardia(VT) while the two “non–shockable” rhythms are asystole and pulseless electrical activity. However, the most important finding of our study is that BCPR can improve the survival of OHCAs whose initial rhythm is shockable rhythm.

So, our meta-analysis was conducted for OHCAs by limiting and no-limiting initial rhythm to show the significance of BCPR and discuss further the mechanism of BCPR improving the survival of OHCAs.

Not surprisingly, when no-limiting the initial rhythm, our results, which BCPR improved the survival of OHCAs [35], were the same as those of previous studies in animals and studies including registry data. Its mechanism, which demonstrated by many epidemiological surveys [29, 36–39], may be prolonging the time window for defibrillation to maintain more shockable rhythm as the initial rhythm when EMS arrived, since the outcome of OHCA is strongly related to whether or not the patient has shockable rhythms when the EMS arrives [16, 40].

When limiting the initial rhythm, our results demonstrated that BCPR still had a good survival compared with NO-BCPR when the initial rhythm of OHCAs was shockable. So we assumed that BCPR not only prolonged the shockable rhythm, but also played other roles for OHCAs. It is possible to keep more cardiomyocytes alive while maintaining the initial rhythm.

For the patients whose initial rhythm is non-shockable, the result of this meta-analysis should be made a further confirmation by more researches and epidemiological surveys.

Some previous articles have demonstrated that BCPR has no effect on patients whose initial rhythm is shockable when EMS arrived. The results of our meta-analysis denied this conclusion and showed BCPR’s effect on VT/VF. Three-phase model of ventricular fibrillation [41] also showed that in the circulatory phase, chest compression could improve the blood supply of myocardium and it took priority over defibrillation. In a word, CPR also has positive effect on the patients whose initial rhythm is VT/VF.

Through multiple meta-regression analysis we concluded that the region was a factor that affects heterogeneity. Previous epidemiological survey [3] also showed that North America had the highest incidence of resuscitation for cardiac arrest and Asia had the lowest percentage of VF and the lowest survival rates. Comparing with America and Europe, popularizing BCPR in Asia is relatively late, so the quality of BCPR might be lower in Asia. And different emergency medical system in different country might also be a factor. Therefore, good experiences from America and Europe should be learned by Asia and other regions. For example, in terms of CPR training, “cascade” training [18] which means instructor trainers train instructors who then train rescuers in their turn might be a good method to make more people accountable, video-based training [42] might be a good way to transmit the right message to more people and easy to deliver, and making CPR training become mandatory might be helpful too. As for increasing the incidence of BCPR, telemedicine and dispatcher assisted CPR [43, 44] might be an effective method.

Because of the simplicity and non-invasive of standard CPR, it is not just a medical practice but a common practice for everyone. Since BCPR has an irreplaceable role in improving the survival of OHCAs, we should pay more attention to increasing the incidence of cardiopulmonary resuscitation and ensuring the quality of cardiopulmonary resuscitation.

A major strength of our study is that this is the first systematic review/meta-analysis which evaluates the effect of BCPR by limiting and no-limiting initial rhythm. Moreover, the number of participants was a relatively large sample size to clarify and confirm this issue. In addition, we strictly enforce the literature search, data extraction and analysis, in order to ensure the credibility of our results.

## Limitations

However, several limitations should be acknowledged. The first is the substantial heterogeneity among included articles. The organization of each country’s emergency system is different, so that the first aid measure provided by them is also different. And the quality of BCPR in each region is uneven due to the different degree of training and emphasis. Some studies suggest that high quality BCPR by professionals is more effective, and ventricular fibrillation as initial rhythm is more common than ordinary bystanders [21]. Nevertheless, in the majority of studies in this meta-analysis, no data were available whether the bystander had been trained in cardiopulmonary resuscitation or not, so we were unable to measure the quality of the BCPR. The second is that the articles which are about OHCAs whose initial rhythm is non-shockable rhythm are less, therefore, the authenticity of the result remains to be confirmed.

## Conclusions

Our meta-analysis provides evidence suggesting that BCPR not only plays an important role for OHCAs including the initial rhythm of OHCAs is shockable rhythm when EMS arrived. But also works for patients whose initial rhythm is shockable. However, the findings should be interpreted cautiously in line with the overall methodological quality and potential biases of the included trials. More clinical studies and basic trials are necessary for further evaluation of the effectiveness of BCPR and exploration the mechanism by which BCPR improves the survival.

## Abbreviations

BCPR: Bystander cardiopulmonary resuscitation
CA: Cardiac arrest
CI: Confidence interval
CPR: Cardiopulmonary resuscitation
ECG: Electrocardiograph
EMS: Emergency medical services
MOOSE: Meta-Analysis of Observational Studies in Epidemiology
NOS: Newcastle-Ottawa Scale
NSFC: Natural Science Foundation of China
OHCAs: Out-of-hospital cardiac arrests
OR: Odds ratio
PICOS: Patients-Intervention-Comparison-Outcome-Study Style
VF: Ventricular fibrillation
VT: Ventricular tachycardia

## References

[1] Kuisma M, Alaspaa A (1997). Out-of-hospital cardiac arrests of non-cardiac origin. Epidemiology and outcome. Eur Heart J.

[2] Sasson C, Rogers MA, Dahl J, Kellermann AL (2010). Predictors of survival from out-of-hospital cardiac arrest: a systematic review and meta-analysis. Circulation Cardiovascular quality and outcomes.

[3] Berdowski Jocelyn, Berg Robert A., Tijssen Jan G.P., Koster Rudolph W. (2010). Global incidences of out-of-hospital cardiac arrest and survival rates: Systematic review of 67 prospective studies. Resuscitation.

[4] Nishiyama C, Brown SP, May S, Iwami T, Koster RW, Beesems SG, Kuisma M, Salo A, Jacobs I, Finn J, Sterz F, Nurnberger A, Smith K, Morrison L, Olasveengen TM, Callaway CW, Shin SD, Grasner JT, Daya M, Ma MH, Herlitz J, Stromsoe A, Aufderheide TP, Masterson S, Wang H, Christenson J, Stiell I, Davis D, Huszti E, Nichol G (2014). Apples to apples or apples to oranges? International variation in reporting of process and outcome of care for out-of-hospital cardiac arrest. Resuscitation.

[5] Leong BS (2011). Bystander cpr and survival. Singap Med J.

[6] Adielsson A, Hollenberg J, Karlsson T, Lindqvist J, Lundin S, Silfverstolpe J, Svensson L, Herlitz J (2011). Increase in survival and bystander cpr in out-of-hospital shockable arrhythmia: Bystander cpr and female gender are predictors of improved outcome Experiences from sweden in an 18-year perspective. Heart (British Cardiac Society).

[7] American Heart A. Guidelines for cardiopulmonary resuscitation (cpr) and emergency cardiac care (ecc) (1974). JAMA.

[8] Stroup DF, Berlin JA, Morton SC, Olkin I, Williamson GD, Rennie D, Moher D, Becker BJ, Sipe TA, Thacker SB (2000). Meta-analysis of observational studies in epidemiology: a proposal for reporting. Meta-analysis of observational studies in epidemiology (moose) group. JAMA.

[9] Higgins JP, Thompson SG (2002). Quantifying heterogeneity in a meta-analysis. Stat Med.

[10] Higgins J. P T (2003). Measuring inconsistency in meta-analyses. BMJ.

[11] Egger M., Smith G. D., Schneider M., Minder C. (1997). Bias in meta-analysis detected by a simple, graphical test. BMJ.

[12] Begg CB, Mazumdar M (1994). Operating characteristics of a rank correlation test for publication bias. Biometrics.

[13] Lund I, people SAC r b l (1976). Lancet (London, England).

[14] Eisenberg M, Bergner L, Hallstrom A (1979). Paramedic programs and out-of-hospital cardiac arrest: I. factors associated with successful resuscitation. Am J Public Health.

[15] Guzy PM, Pearce ML, Greenfield S (1983). The survival benefit of bystander cardiopulmonary resuscitation in a paramedic served metropolitan area. Am J Public Health.

[16] Roth R, Stewart RD, Rogers K, Cannon GM (1984). Out-of-hospital cardiac arrest: factors associated with survival. Ann Emerg Med.

[17] Weston CF, Wilson RJ, Jones SD (1997). Predicting survival from out-of-hospital cardiac arrest: a multivariate analysis. Resuscitation.

[18] Holmberg M, Holmberg S, Herlitz J (2000). Effect of bystander cardiopulmonary resuscitation in out-of-hospital cardiac arrest patients in Sweden. Resuscitation.

[19] Herlitz J, Bang A, Gunnarsson J, Engdahl J, Karlson BW, Lindqvist J, Waagstein L (2003). Factors associated with survival to hospital discharge among patients hospitalised alive after out of hospital cardiac arrest: change in outcome over 20 years in the community of goteborg, Sweden. Heart.

[20] Herlitz J, Svensson L, Holmberg S, Angquist KA, Young M (2005). Efficacy of bystander cpr: intervention by lay people and by health care professionals. Resuscitation.

[21] Nordberg P, Hollenberg J, Herlitz J, Rosenqvist M, Svensson L (2009). Aspects on the increase in bystander cpr in Sweden and its association with outcome. Resuscitation.

[22] Lindner TW, Søreide E, Nilsen OB, Torunn MW, Lossius HM (2011). Good outcome in every fourth resuscitation attempt is achievable-an utstein template report from the Stavanger region. Resuscitation.

[23] Wissenberg M, Folke F, Weeke P, Hansen CM, Olesen J, Lindhardsen J, Lippert F, Gislason G, Nielsen SL, Kober L, Torp-Pedersen C (2012). Improved survival and increase in bystander cardiopulmonary resuscitation after outof-hospital cardiac arrest: a nationwide cohort study 2001-2010. J Am Coll Cardiol.

[24] Hasselqvist-Ax I, Riva G, Herlitz J, Rosenqvist M, Hollenberg J, Nordberg P, Ringh M, Jonsson M, Axelsson C, Lindqvist J, Karlsson T, Svensson L (2015). Early cardiopulmonary resuscitation in out-of-hospital cardiac arrest. N Engl J Med.

[25] Iwami T, Kawamura T, Hiraide A, Berg RA, Hayashi Y, Nishiuchi T, Kajino K, Yonemoto N, Yukioka H, Sugimoto H, Kakuchi H, Sase K, Yokoyama H, Nonogi H (2007). Effectiveness of bystander-initiated cardiac-only resuscitation for patients with out-of-hospital cardiac arrest. Circulation.

[26] Lai H, Choong CV, Fook-Chong S, Ng YY, Finkelstein EA, Haaland B, Goh ES, Leong BS, Gan HN, Foo D, Tham LP, Charles R, Ong ME (2015). Interventional strategies associated with improvements in survival for out-of-hospital cardiac arrests in Singapore over 10 years. Resuscitation.

[27] Tanaka H, Ong MEH, Siddiqui FJ, Ma MHM, Kaneko H, Lee KW, Kajino K, Lin CH, Gan HN, Khruekarnchana P, Alsakaf O, Rahman NH, Doctor NE, Assam P, Shin SD (2017). Modifiable factors associated with survival after out-of-hospital cardiac arrest in the pan-asian resuscitation outcomes study. Annals of emergency medicine.

[28] Cummins RO, Eisenberg MS, Hallstrom AP, Litwin PE (1985). Survival of out-of-hospital cardiac arrest with early initiation of cardiopulmonary resuscitation. Am J Emerg Med.

[29] Swor RA, Jackson RE, Cynar M, Sadler E, Basse E, Boji B, Rivera-Rivera EJ, Maher A, Grubb W, Jacobson R (1995). Bystander cpr, ventricular fibrillation, and survival in witnessed, unmonitored out-of-hospital cardiac arrest. Ann Emerg Med.

[30] Stiell IG, Wells GA, DeMaio VJ, Spaite DW, Field BJ, Munkley DP, Lyver MB, Luinstra LG, Ward R (1999). Modifiable factors associated with improved cardiac arrest survival in a multicenter basic life support/defibrillation system: opals study phase i results. Ontario prehospital advanced life support. Ann Emerg Med.

[31] Gaieski DF, Agarwal AK, Abella BS, Neumar RW, Mechem C, Cater SW (2017). Adult out-of-hospital cardiac arrest in Philadelphia from 2008–2012: an epidemiological study. Resuscitation.

[32] Finn JC, Jacobs IG, Holman CD, Oxer HF (2001). Outcomes of out-of-hospital cardiac arrest patients in Perth, Western Australia, 1996-1999. Resuscitation.

[33] Nehme Z, Bernard S, Cameron P, Bray J, Meredith I, Lijovic M, Smith K (2015). Using a cardiac arrest registry to measure the quality of emergency medical service care: a decade of findings from the victorian ambulance cardiac arrest registry. EMA - Emergency Medicine Australasia.

[34] Herlitz J, Svensson L, Engdahl J, Silfverstolpe J (2008). Characteristics and outcome in out-of-hospital cardiac arrest when patients are found in a non-shockable rhythm. Resuscitation.

[35] Kitamura T, Iwami T, Kawamura T, Nitta M, Nagao K, Nonogi H, Yonemoto N, Kimura T (2012). Nationwide improvements in survival from out-of-hospital cardiac arrest in Japan. Circulation.

[36] Waalewijn RA, Nijpels MA, Tijssen JG, Koster RW (2002). Prevention of deterioration of ventricular fibrillation by basic life support during out-of-hospital cardiac arrest. Resuscitation.

[37] Christenson J, Andrusiek D, Everson-Stewart S, Kudenchuk P, Hostler D, Powell J, Callaway CW, Bishop D, Vaillancourt C, Davis D, Aufderheide TP, Idris A, Stouffer JA, Stiell I, Berg R (2009). Chest compression fraction determines survival in patients with out-of-hospital ventricular fibrillation. Circulation.

[38] Ritter G, Wolfe RA, Goldstein S, Landis JR, Vasu CM, Acheson A, Leighton R, Medendrop SV (1985). The effect of bystander cpr on survival of out-of-hospital cardiac arrest victims. Am Heart J.

[39] Herlitz J, Ekstrom L, Wennerblom B, Axelsson A, Bang A, Holmberg S (1996). Type of arrhythmia at ems arrival on scene in out-of-hospital cardiac arrest in relation to interval from collapse and whether a bystander initiated cpr. Am J Emerg Med.

[40] Gilmore CM, Rea TD, Becker LJ, Eisenberg MS (2006). Three-phase model of cardiac arrest: time-dependent benefit of bystander cardiopulmonary resuscitation. Am J Cardiol.

[41] Vilke GM, Chan TC, Dunford JV, Metz M, Ochs G, Smith A, Fisher R, Poste JC, McCallum-Brown L, Davis DP (2005). The three-phase model of cardiac arrest as applied to ventricular fibrillation in a large, urban emergency medical services system. Resuscitation.

[42] Bystander cpr (2005). A new video-based training program delivers the right message to the right people. JEMS.

[43] Atkinson PR, Bingham J, McNicholl BP, Loane MA, Wootton R (1999). Telemedicine and cardiopulmonary resuscitation: the value of video-link and telephone instruction to a mock bystander. J Telemed Telecare.

[44] Bang A, Ortgren PO, Herlitz J, Wahrborg P (2002). Dispatcher-assisted telephone cpr: a qualitative study exploring how dispatchers perceive their experiences. Resuscitation.

